# Magnetoencephalography

**DOI:** 10.1136/practneurol-2013-000768

**Published:** 2014-03-19

**Authors:** Malcolm Proudfoot, Mark W Woolrich, Anna C Nobre, Martin R Turner

**Affiliations:** 1 Nuffield Department of Clinical Neurosciences, University of Oxford, UK; 2 Oxford Centre for Human Brain Activity, University of Oxford, UK

**Keywords:** NEUROPHYSIOLOGY, EXPT, BRAIN MAPPING

## Introduction

The understanding of brain function is moving rapidly towards a systems-level, network-based approach. It is now as naive to talk simplistically about what a particular area of the brain ‘does’, as it was for Franz Joseph Gall (1758–1828) to link its performance to the thickness of the overlying skull. Magnetoencephalography (MEG) is a rapidly developing and unique tool for the study of brain function, in particular the underlying oscillations in neuronal activity that appear to be fundamental (box 1), with real-time resolution and potential for application across a range of brain disorders. We provide a brief overview of the technology, broad approaches to data analysis, and aspirations for its application to the study of neurodegeneration.

Box 1 **An introduction to neuronal oscillations**

- Neuronal oscillatory activity is continuous, but fluctuations in power and timing allow rapid alteration in communication strength within existing structural network architecture, far faster than synaptic modification.^1^
- Two distinct cerebral regions can facilitate preferential information exchange by synchronising their rhythmic behaviour; the γ band (40–80 Hz), in particular, facilitates this process, but is also modulated ‘top-down’ by lower frequencies such as θ (4–7 Hz), reflecting factors, such as arousal states.
- α Rhythms (8–13 Hz), so prominent in the occipital cortex upon eye closure, reflect more than just an ‘idling’ rhythm but also contribute to active allocation of attentional resources and suppress irrelevant sensory information.^2^
- The influential theory ‘Communication through Coherence’ developed by Fries,^3^ builds on existing models of ‘binding by synchronisation’ that may underpin selective attention, a key function in prioritising neural events to guide awareness and action.^4^

## Functional brain imaging so far

Structural MR imaging of the brain and spinal cord has revolutionised the accuracy of diagnosis in common conditions, such as stroke, and greatly expanded the taxonomy of neurological disorders. Advanced applications of MRI now allow the assessment of white matter tract integrity (diffusion-tensor imaging), regional grey matter volume (voxel-based morphometry), and cortical thickness (surface-based morphometry). These techniques enable the non-invasive and rapid quantification of structure at a given time point, but it is clear that the brain cannot be understood in terms of structure alone. *Functional* MRI (fMRI), based on blood oxygen-level-dependent (BOLD) image contrast, can achieve almost submillimetre accuracy in the spatial localisation of neuronal activity. However, this relatively high spatial resolution is not matched in temporal accuracy, in essence because the relatively slow speed of haemodynamic changes in response to neuronal activity fundamentally limits the temporal detail that can be extracted to a timescale of seconds. When one considers the multiple synaptic transmissions and physical distance covered by brain activity within this time frame, it is quickly appreciated that this technology is limited in its ability to deliver a systems-level understanding of brain function if used in isolation.

## The unique advantages of MEG

Ever since the pioneering EEG recordings made by German neurologist Hans Berger (1873–1941), it has been possible to identify the self-generated oscillatory activity of neuronal ensembles and categorise frequency bands with increasing accuracy. Electrical potential changes related to brain activity measured at the scalp by EEG are fundamentally limited by the distortive effects of the intervening structures, which severely hamper efforts to localise the signal source precisely. MEG, instead, measures the magnetic field changes induced by intracellular current flow, the generation of which obeys the ‘right-hand rule’ in the application of Ampère's law. Unlike EEG measures, these pass through dura, skull and scalp relatively unaltered. The technique, therefore, offers a safe, non-invasive method to ‘listen’ in to brain activity at rest and during simple tasks, which from the subject's perspective, despite measuring at several hundred channels, is painless and quick to set up. Mathematical modelling of these data then enables localisation of sources while uniquely maintaining sampling frequencies up to several thousand times per second. Compared to fMRI's temporal resolution of, at best, several hundred milliseconds, MEG can resolve events with millisecond precision.

## How it works

**Figure 1:**
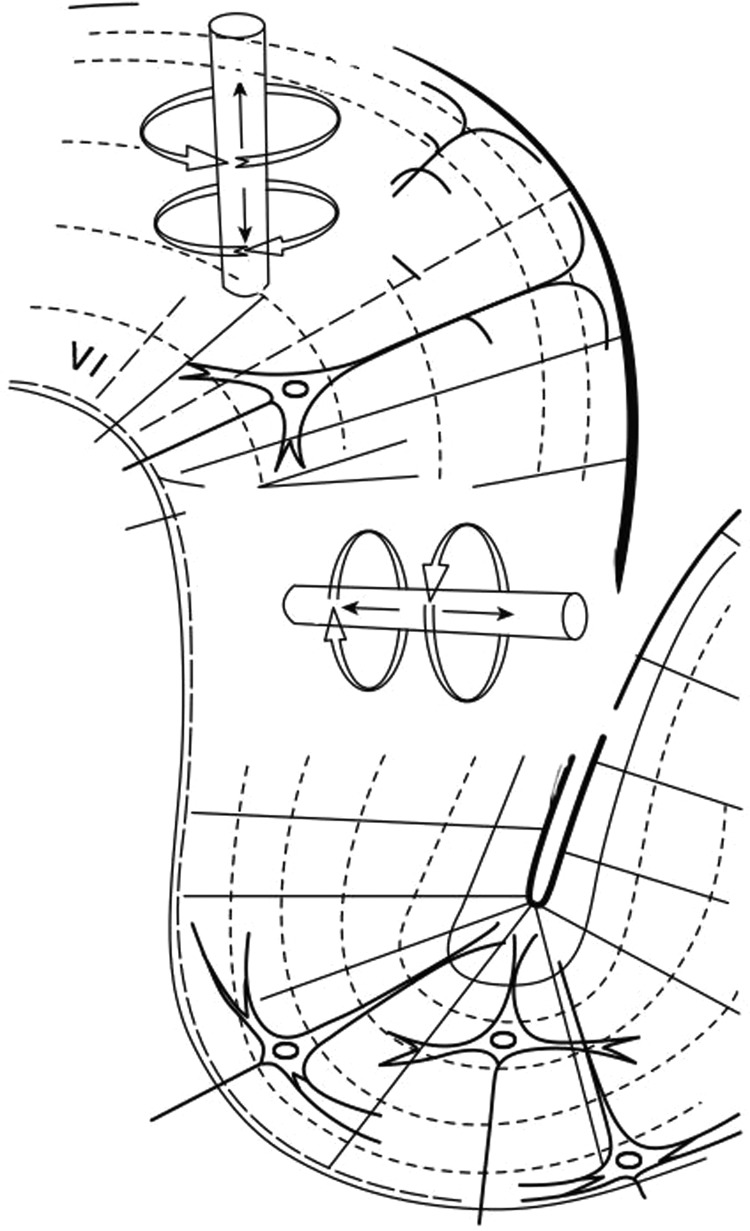
The organisation of cortical microcolumns within the sulcal bank, tangentially orientated to the skull, allows their detection with magnetoencephalography since their induced magnetic fields will project beyond the skull surface. Conversely, apical dendrites orientated perpendicularly to the skull, as found at the gyral crown, are better detected by EEG. (From Hansen *et al*^5^).

The neuronal activity captured by MEG is not, as perhaps expected, generated by the (too brief) axonal action potentials of pyramidal cells, but rather by the net contributions of excitatory and inhibitory dendritic postsynaptic potentials. This current flow through the apical dendrites (represented as a ‘dipole’) generates a magnetic field that projects radially; thus, MEG excels at detecting dipoles arranged in a tangential orientation to the skull. Fortunately, the extensively folded sulci of the human cortex promote that orientation for the majority of cortical microcolumns (figure 1). However, MEG is less sensitive to deeper (including subcortical) sources, as magnetic field change decreases rapidly with distance.

**Figure 2:**
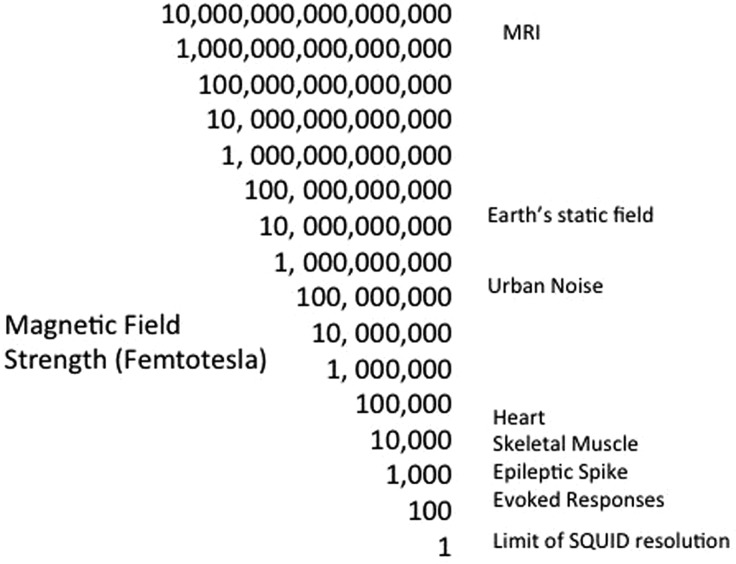
Magnetic field strength density measured in femtotesla (fT), highlighting the exquisite sensitivity of the SQUIDs used in magnetoencephalography.

Compared with a standard clinical MR scanner magnet strength of 1.5 Tesla, the strength of the signals detected by MEG are 10^14^ orders smaller (figure 2). It has been compared with hearing a pin drop at a rock concert. The smallest measurable magnetic field changes are thought to be produced by simultaneously active arrays of approximately 50 000 pyramidal cells, which in theory covers a cortical surface area of 0.9 mm diameter. It is increasingly recognised that modulation of self-generated oscillatory activity is a principal mechanism by which geographically distant network regions interact,^6^ thus, a brain-wide imaging technique with high temporal sensitivity is a prerequisite for interrogation.

**Figure 3:**
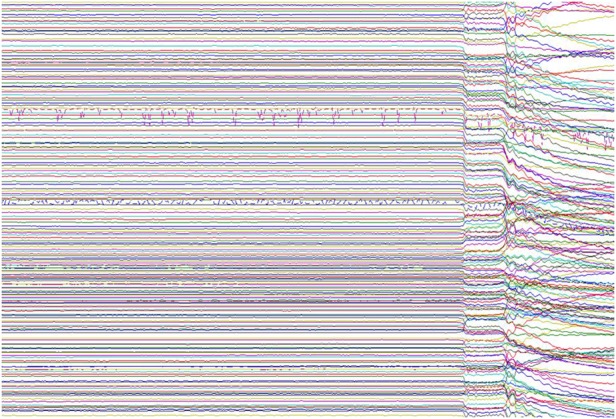
After 4 s of raw magnetoencephalography data (two channels contain obvious artefacts), the door to the magnetically shielded room is opened during recording. The interference caused by external magnetic fields highlights why effective room shielding is essential.

**Figure 4:**
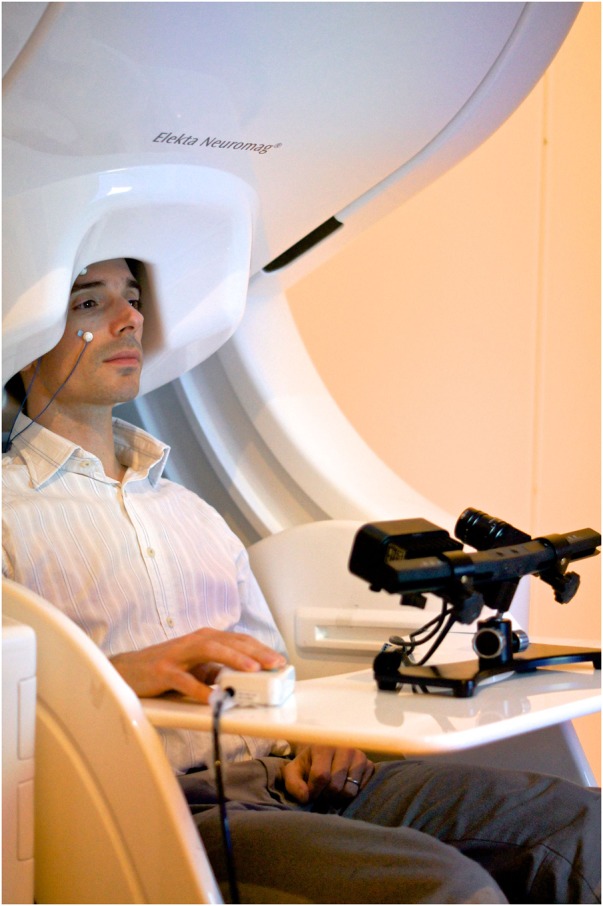
A MEG recording session at OHBA, eyetracker device shown in foreground.

The ability to detect endogenously generated magnetic fields was realised in the 1960s by physicist David Cohen at Massachusetts Institute of Technology, who furthered the then recent discovery of ‘magnetocardiography’ by applying a magnetically shielded room to remove the overwhelming noise of the Earth's magnetic field (figure 3). He could then measure the even smaller magnetoencephalographic signal by making use of superconducting loops superconducting quantum interference device (SQUIDs), developed by his collaborator James Zimmerman. At very low temperatures, SQUIDs are extremely sensitive to magnetic field change, which can be recorded and converted into digital signal (‘quantisation’). Sensor arrays have evolved to provide whole-head coverage via a helmet containing more than 300 sensor sites, enveloped in a ‘dewar’ of cooling liquid helium. The subject's head is positioned underneath the helmet (figure 4) and it is possible to acquire EEG recordings simultaneously if desired. In the future, MEG may include atomic sensors, which still exploit the principle of quantum tunnelling of pairs of electrons, but obviate the need for cryogenic temperatures.^7^

## The acquisition

The sensor design itself has evolved to meet some of the localisation challenges by using more than one pick-up coil in series. A single ‘magnetometer’ coil measures any orthogonal magnetic field. Pairs of coils place closed together and wound in opposite directions are also used to measure gradients in the magnetic field over space. These ‘gradiometers’ are particularly sensitive to a gradient in magnetism from nearby (ie, neuronal) sources, but subtract out signal from distant external (and thus artefactual) sources, as these appear similar to both coils.

Electromagnetic signals are also generated by movement of the head or eyes (including blinking), skeletal and cardiac muscle electromagnetic activity. The sensors, therefore, also pick up this physiological noise, unrelated to brain activity. Interference from dental amalgam, metal zippers, jewellery, and bra fasteners is also significant and avoided by pre-scan screens if possible. Head location must be calculated relative to the SQUID sensors, since the helmet is not a tight fit. This is achieved by using small magnetic coils attached to anatomical landmarks to localise the subject's head position in the MEG scanner, and these can also be used to enable continuous motion correction. However, it is also important to maximise subject comfort to discourage excess head movement.

Considerable care must also be taken in ensuring that all stimulus presentation and response devices within the magnetically shielded room are themselves electromagnetically silent. Experimental tasks are typically designed to avoid eye movements during relevant measurement periods in order to minimise artefact, but remaining eye movements and blinks can still be identified with a combination of surface electro-oculography and infrared eye tracking. An ECG is typically also recorded to enable identification and subsequent exclusion of cardiac electrical activity, which can otherwise create large contaminating artefacts in the MEG signal. There are comprehensive expert consensus guidelines aiming to harmonise MEG experimental strategies across sites^8^ in an effort to improve reproducibility.

## The analysis

MEG analysis involves enormous datasets that require vast computer processing power to manipulate them. Having overcome the initial difficulties in artefact identification, MEG data analysis still involves considerable complexity. The fundamental issue is that of the ‘inverse problem’. This concept, in relation to MEG, summarises the challenge of precisely localising in three dimensional (3D) space the underlying neural sources of a magnetic recording. In reality, there may be many equally plausible combinations of neural sources, and thus, without additional constraints the solution is not unique. Yet, this is a challenge our own brains overcome daily by using constraints that reflect sensible prior assumptions, for example in deciding if a visual object is small and close, or large and far away. We have existing expectations about object size to guide us.

Methods to solve the inverse problem, therefore, need to make additional assumptions, such as the brain activity being spatially sparse or smooth. The appropriate assumptions to make are often directed by the particular experimental protocol and expected findings. A given paradigm may seek to identify differential brain activity during a rudimentary sensory stimulation task, in which case the localisation model might assume that a small number of dipole sources could account for the difference in signal production (ie, the brain activity is sparse). Alternatively, for example during cognitive tasks, there is a wide net of possible sources in the brain to consider, and we need more advanced modelling techniques. This includes approaches that assume the brain activity is smooth and sparse,^9^ and also constrained to a 3D map of the patient's own cortical mantle (obtained during a subsequent structural MRI). A common approach for representing these various assumptions is to use priors in powerful Bayesian algorithms. A popular alternative to overcome the inverse problem is to use beamforming. This method, originally developed for use in radar arrays, corresponds to an adaptive spatial filter designed to extract the origins of a signal from some prespecified spatial location.^10^

**Figure 5:**
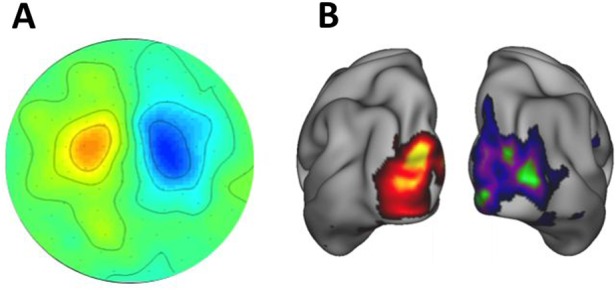
Sensor space MEG data (A) presented as a two dimensional (2D) topographic map of contrasted α (8–12 Hz) activity in a visual attention task (data concatenated from 38 subjects). α power is lower in the contralateral (attending) hemisphere. MEG data reconstructed into source space (B) with a beamformer approach and presented on a 3D cortical map.

MEG data in either ‘sensor space’ (presented as data recorded across the distribution of the sensors) or ‘source space’ (reconstructed to a 3D model of brain sources) offer a wealth of analysis possibilities and selected features that can relate to a particular clinical question (figure 5).

**Figure 6:**
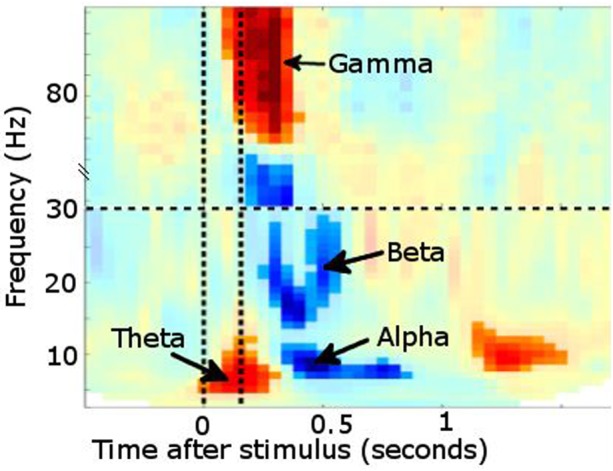
Time–frequency plot from posterior sensors following presentation of a visual stimulus (multiple subjects combined). Increased power is noted in θ followed shortly by γ. Desynchronisation instead occurs in α and β bands. Vertical lines denote stimulus onset/offset.

For example, it is possible to analyse the data time-locked to experimental events in terms of event-related fields (ERF, the MEG analogue of event-related potentials), such as the mismatch negativity abnormalities described in dyslexia.^11^ Additionally, MEG is particularly powerful at investigating oscillatory brain activity by using ‘Fourier transformations’, or related transforms, such as Wavelet or Hilbert, to describe the signal in terms of how it oscillates in different frequency bands over time. These frequency bands show meaningful changes in power or synchronisation of ongoing oscillatory activity at behaviourally relevant time points, and can also form the basis of functional connectivity measurements (correlated changes over time) between different brain regions captured in MEG data^1^,^2^ (figure 6).

## The applications and potential of MEG

**Figure 7:**
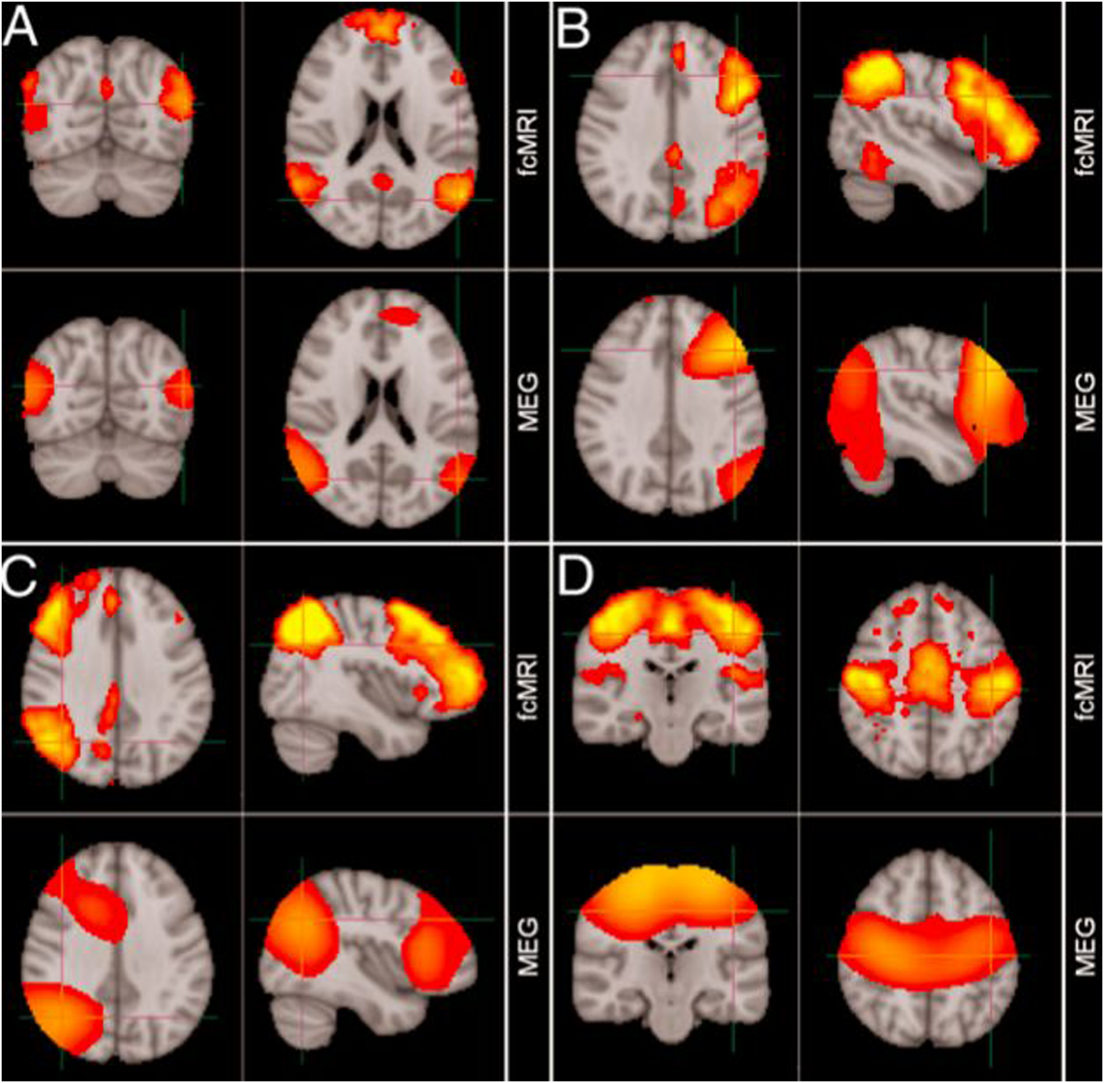
Comparison of resting-state networks identified from magnetoencephalography and fMRI data independently. (A) Default mode network, (B) left lateral frontoparietal network, (C) right lateral frontoparietal network, (D) sensorimotor network. (Adapted from Brookes *et al*^14^).

MEG has found early clinical application in epileptic source localisation, in particular for the planning of subsequent surgery, and shows close correlation to invasive studies of cortical activity.^13^ In a new era, MEG applications are no longer limited to description of relatively fundamental neurophysiology, but have instead begun to describe whole-brain activity at the network-level during the so-called resting state^14^ (figure 7) as well as task performance.^15^

This approach has been pioneered by fMRI,^16^ but MEG now validates these findings in eliminating artefactual explanations based solely on correlated patterns of vascular activity. MEG also offers the possibility of decomposing the time-course of communication between ‘nodes’ in such networks, assessing their functionality during task activity, and indeed describing their dysfunction during neurodegenerative disease,^17^ with the ultimate hope of developing sensitive pharmacodynamic markers of therapeutic response. MEG also offers information complementary to fMRI; the uncertain impact of disease on the time course of the BOLD haemodynamic response function encourages multimodal data acquisition during brain activation (by necessity non-concurrent, as MEG and fMRI cannot be acquired simultaneously).^18^

### MEG correlates of neurodegeneration

Focal slowing in temporoparietal regions was an expected finding in early MEG studies of Alzheimer's disease, and was furthermore correlated with cognitive measures.^19^ Attempts to model the source of spontaneous α band activity also noted an anterior shift from parieto-occipital regions to predominantly temporal-lobe generators in Alzheimer's disease versus controls; this was interpreted to reflect somehow a loss of cholinergic transmission.^20^ Another study provided more convincing evidence that described left temporal MEG activity deficits, which correlated with ipsilateral hippocampal atrophy and behavioural measures.^21^

Surface EEG had already described abnormalities in preattentive auditory processing of deviant tones in neurodegenerative disease. These excessive evoked response potential (ERP) abnormalities, perhaps reflecting a failure to inhibit or adapt to irrelevant stimuli, were convincingly replicated and localised in MEG studies of Parkinson's disease,^22^ Alzheimer's disease^23^ and amyotrophic lateral sclerosis.^24^

Subjects with Parkinson's disease, even without dementia, also show widespread oscillatory slowing.^25^ Furthermore, in combination with invasive local recordings, MEG identified a dopamine-responsive long-distance network operating in the β band that showed coherence between prefrontal cortex and subthalamic nuclei, in contrast to a spatially and spectrally distinct network in the α band connecting brainstem with temporoparietal cortex.^26^

### Global resting-state networks in neurodegeneration

A growing number of MEG studies have described neurodegenerative alteration to functional connectivity during resting-state recordings, complementing findings from fMRI. Resting-state networks describe the temporally coordinated, but often anatomically disparate, spontaneous fluctuations in brain activity that are present even at rest within distinct functional brain networks.^27^ Although such networks are obviously engaged during relevant task activity, study of resting-state networks is particularly appealing within patient populations as impaired task performance is no longer a possible confounding factor.

Alzheimer's disease has been most frequently scrutinised using resting-state measures. A study of 18 patients (mean Mini-Mental State Examination Score 19.2) noted a loss of long-distance interhemispheric connectivity (measured as synchronisation likelihood) within the α and β bands. This correlated with cognitive impairment and was not consequent to anticholinesterase treatment.^28^ A repeat study focused on the modular organisation of resting-state networks (decomposed into functional subnetworks), and noted Alzheimer's disease to cause decrement of *intermodular* connectivity but also *intramodular* damage restricted to certain cortical regions.^29^

Such graph-theory concepts could, in principle, be employed to transfer descriptions of a ‘connectome’ (our unique neural fingerprint that defines us as individuals) across different modalities, including fMRI and diffusion tensor imaging (DTI) studies.^30^ However, results from these connectivity studies often conflict, perhaps reflecting differing population samples, but also implying a mismatch between functional and structural connection strength,^31^ which may hint at underlying pathological mechanisms, such as interneuronal dysfunction in amyotrophic lateral sclerosis.^32^

### Oscillatory signatures in neurodegeneration

The suitability of MEG to capture neuronal activity during relevant motor or cognitive tasks has encouraged parallel investigation of induced changes in oscillatory activity—perturbations of spontaneous brain activity patterns are fundamental to all our experiences, decisions and actions.^1^ A block-design study of patients with Alzheimer's disease or dementia with Lewy bodies contrasted their cortical spectral power during periods of either rest or repeated auditory attention tasks. In both conditions, there were large group differences from anterior sensors in a 3–7 Hz θ band.^33^ An event-related experimental design was used to study visual working memory tasks attempted by Alzheimer's disease and vascular dementia patients.^34^ Those dementia subjects still able to complete the task successfully demonstrated (against controls) significantly delayed and stronger amplitude α desynchronisation during the task epochs (albeit with limited topographical localisation), in keeping with the comparatively higher burden of cognitive process.

Task-based MEG recordings of 12 subjects with frontotemporal dementia were compared with controls, while making categorical semantic judgements about visually presented objects. There was an early difference in temporoparietal cortex activity followed by a later reduction in frontoparietal activity in each task epoch, interpreted as corresponding to semantic information processing and subsequent action selection.^35^ This result highlights the high temporal resolution achievable in MEG recordings such that component parts of a rapid cognitive task can be distinguished with confidence in time and anatomical space.

Even swallowing has proved to be an informative motor task in the study of neurodegenerative disease with MEG. An investigation of Parkinson's disease subjects with and without dysphagia found evidence for compensatory adaptive cerebral changes to involve wider cortical regions.^36^ The same researchers have noted right hemispheric lateralisation of swallow-related activity in amyotrophic lateral sclerosis^37^ and Kennedy's syndrome,^38^ perhaps reflecting plasticity in the face of progressive neurodegeneration.

### Biomarker potential

MEG has been used to predict whether subjects were more likely to progress to clinically defined Alzheimer's disease on the basis of more widespread power changes during a memory task, in a study of a small number of subjects with minimal cognitive impairment.^39^ A larger (117 Alzheimer's disease subjects) multicentre MEG study was subsequently performed; 1 min of resting-state data was sufficient to detect increased functional connectivity.^40^ Ten-month interval assessment of 31 subjects detailed progressive abnormalities, correlating with worsening neuropsychometry. A longitudinal study of Parkinson's disease showed progressive slowing of oscillatory activity over a 4-year follow-up period in 59 initially non-demented Parkinson's disease subjects.^41^ These changes were particularly associated with mild cognitive decline and, furthermore, MEG signals, above and beyond neuropsychometry, predicted subsequent conversion to Parkinson's disease dementia, as borne out at the 7-year follow-up time point.^42^

## Conclusion

In combination with the increasingly high structural resolution offered by MRI, MEG has unique potential as part of a multimodal approach to brain disorders that is sensitive to time as well as space. It seems clear from presymptomatic studies across a range of neurodegenerative disorders, that the earliest events occur many years, possibly decades, before the onset of symptoms. Primary prevention of neurodegeneration will require sensitivity to very subtle changes in brain activity that seem likely to operate at the network level.^43^ As well as studying the patterns of brain activity in the resting state, cognitive or motor MEG-based tasks could reveal key pathological or compensatory patterns of activity that might form the basis for early intervention, including pharmacodynamic biomarkers of therapeutic intervention.

**Key points**

- Magnetoencephalography (MEG) is non-invasive, safe and comfortable.
- MEG offers unsurpassed temporal resolution; it can probe neuronal oscillation activity that is fundamental to brain function in health and disease.
- Modern MEG systems include several hundred distortion-free sensors surrounding the head to provide high spatial precision over superficial cortical areas.
- MEG analysis describes in vivo function of whole brain networks in real time, and has the potential to detect the very earliest changes in neurodegenerative disorders and assess preventative therapies of the future.
